# Novel Approach to Dressing Using Vinegar-Soaked Gauze and Grated Papaya in the Management of Pseudomonas-Infected Pressure Ulcers: A Case Report

**DOI:** 10.7759/cureus.91157

**Published:** 2025-08-28

**Authors:** Parin Y Patel, Ronak Rathod

**Affiliations:** 1 General Surgery, Health 1 Super Speciality Hospital, Ahmedabad, IND

**Keywords:** enzymatic debridement, low-resource setting, natural therapies, papaya enzymes, pressure ulcer, pseudomonas infection, vinegar dressing, wound care

## Abstract

Pressure ulcers in chronically immobilized patients often become infected with multidrug-resistant organisms such as Pseudomonas aeruginosa. Management is particularly challenging in low-resource settings where advanced wound care products are unavailable. Natural, inexpensive alternatives, such as vinegar and papaya, have demonstrated antimicrobial and debriding properties, but their combined use has not been formally documented.

A 65-year-old male with advanced Parkinsonian disease and a prior cerebrovascular accident, bedridden and dependent for care, presented with multiple Stage III-IV gluteal pressure ulcers. The largest measured approximately 25 × 25 cm with a depth of 2 cm, all with foul odor, greenish discharge, and heavy slough. Pseudomonas aeruginosa infection was confirmed on wound culture. Initial povidone-iodine dressings for one week failed to improve the ulcers. A novel regimen was then introduced: freshly grated unripe papaya was applied directly to the wound bed, followed by sterile gauze soaked in 5% white vinegar. Dressings were performed twice daily initially, then once daily as the wound improved. Within 3 days, odor and green staining had markedly reduced; by day 5, approximately 70% of slough had cleared; and by day 10, the wounds demonstrated a clean, granulating base. Once the ulcers reached an inflamed “angry wound” stage, papaya-vinegar applications were discontinued, and saline dressings were recommended. The patient tolerated the regimen well, reporting only transient burning with vinegar application. Unfortunately, he died at home soon after, unrelated to the ulcers, precluding long-term follow-up.

Grated papaya combined with vinegar dressing may provide a low-cost option for managing Pseudomonas-infected pressure ulcers in resource-limited settings. This approach facilitated rapid odor reduction, slough clearance, and granulation when conventional dressings failed, but requires discontinuation once the wound becomes inflamed. Further studies are needed to establish efficacy and safety.

## Introduction

Pressure ulcers, also referred to as decubitus ulcers, are common complications in elderly, paralyzed, or chronically immobilized patients. These lesions typically result from prolonged pressure, shear forces, and compromised tissue perfusion, most often developing over bony prominences such as the sacrum or gluteal region. In many cases, especially in developing countries, delayed presentation and limited access to specialized care contribute to chronic, non-healing ulcers that are prone to secondary infections. Neurological conditions, such as advanced Parkinson’s disease or stroke, further increase vulnerability by rendering patients immobile and dependent on caregivers, while poor nutritional support exacerbates impaired wound healing [1].

Among the various pathogens implicated in chronic wound infections, Pseudomonas aeruginosa remains one of the most challenging. This gram-negative bacillus is well-known for its multidrug resistance and biofilm-forming (structured bacterial communities that protect bacteria from antibiotics and host defenses) ability, which severely impairs antibiotic penetration and delays healing [2]. Clinically, Pseudomonas-infected wounds are characterized by greenish discoloration, heavy exudate, and a distinct pungent odor, which can cause both local tissue damage and psychological distress for patients and caregivers [3].

Standard management of pressure ulcers involves pressure offloading, debridement (removal of necrotic tissue), application of topical antiseptics or antibiotics, and systemic antimicrobial therapy when necessary. However, advanced wound care modalities, such as silver-impregnated dressings, hydrocolloids, enzymatic debriders (agents that chemically break down slough and necrotic tissue), and negative pressure wound therapy, are frequently unavailable or unaffordable in low-resource healthcare settings [4]. These limitations have prompted interest in the use of natural, affordable, and locally accessible agents.

Acetic acid (vinegar), commonly available in household and clinical settings at concentrations of 1-5%, has demonstrated antibacterial activity against Pseudomonas aeruginosa and other gram-negative organisms, largely through its ability to disrupt bacterial membranes and dissolve biofilms [5]. Similarly, unripe papaya is rich in proteolytic enzymes, particularly papain and chymopapain, which assist in the enzymatic breakdown of necrotic tissue and facilitate wound bed preparation. This helps create conditions favorable for the development of granulation tissue (new connective tissue and capillaries that form during healing) [6]. Traditional medicine in tropical regions has long recognized papaya’s role in wound cleansing and debridement.

In our prior clinical experience, this dressing method had been informally attempted in different hospital settings and consistently produced encouraging results, though these instances were not formally documented or published. The present report, therefore, represents the first structured documentation of a papaya-vinegar dressing in the management of Pseudomonas-infected pressure ulcers.

We report a case in which these two agents were used in combination as a novel dressing technique for managing severe gluteal pressure ulcers in a paralyzed elderly patient.

## Case presentation

A 65-year-old male with advanced Parkinsonian disease (Hoehn and Yahr stage V, functionally immobile) and a past history of cerebrovascular accident was brought to the outpatient surgical clinic by his relatives with complaints of multiple non-healing ulcers over the gluteal region. He had been bedridden for several weeks, entirely dependent on caregivers for all activities of daily living. At home, he was nursed on a water bed but lacked regular repositioning protocols and received no nutritional supplementation. His other systemic comorbidities were controlled, and there were no signs of systemic sepsis, such as fever, tachycardia, or leukocytosis, at presentation.

**Figure 1:**
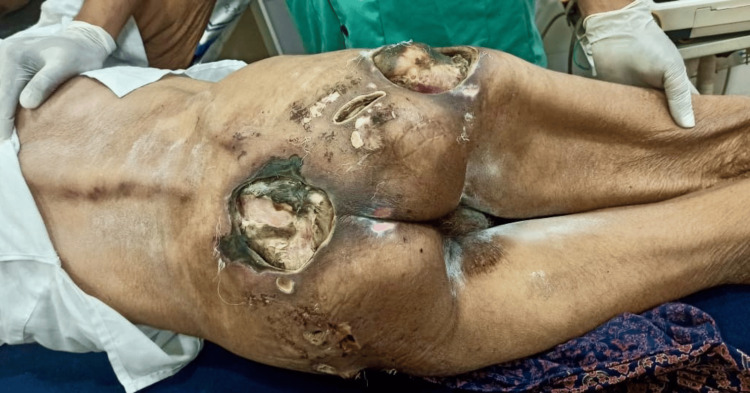
Initial presentation showing extensive gluteal pressure ulcers with thick yellow slough, greenish discoloration of tissue, and foul discharge consistent with Pseudomonas aeruginosa infection

On local examination, three large pressure ulcers were identified bilaterally over the buttocks, along with several smaller satellite lesions. The largest, located over the right gluteal prominence, measured approximately 25 × 25 cm with a depth of nearly 2 cm. It had irregular, undermined edges, thick yellow slough, and purulent, foul-smelling discharge, consistent with a Stage IV pressure ulcer. The second ulcer, measuring about 18 × 15 cm, with a depth of 1.5 cm, demonstrated necrotic tissue and purulent greenish exudate, also consistent with Stage IV disease. The third lesion, on the contralateral side, measured approximately 12 × 10 cm with a depth of 1.5 cm, with slough and necrotic patches, compatible with a Stage III-IV ulcer. Several smaller satellite ulcers ranging from 5 to 10 cm in diameter were scattered bilaterally across the gluteal surface (Figure 1).

All wounds emitted a strong malodor, and gauze dressings quickly became green-stained. Based on these findings, Pseudomonas aeruginosa infection was suspected and subsequently confirmed by wound swab culture.

Initial management consisted of superficial debridement of necrotic tissue to allow drainage of underlying pus, followed by daily packing of the ulcers with povidone-iodine-soaked gauze. Despite consistent dressings for one week, there was no improvement: the ulcers remained sloughy and malodorous, and the gauze continued to turn green within hours of application.

**Figure 2:**
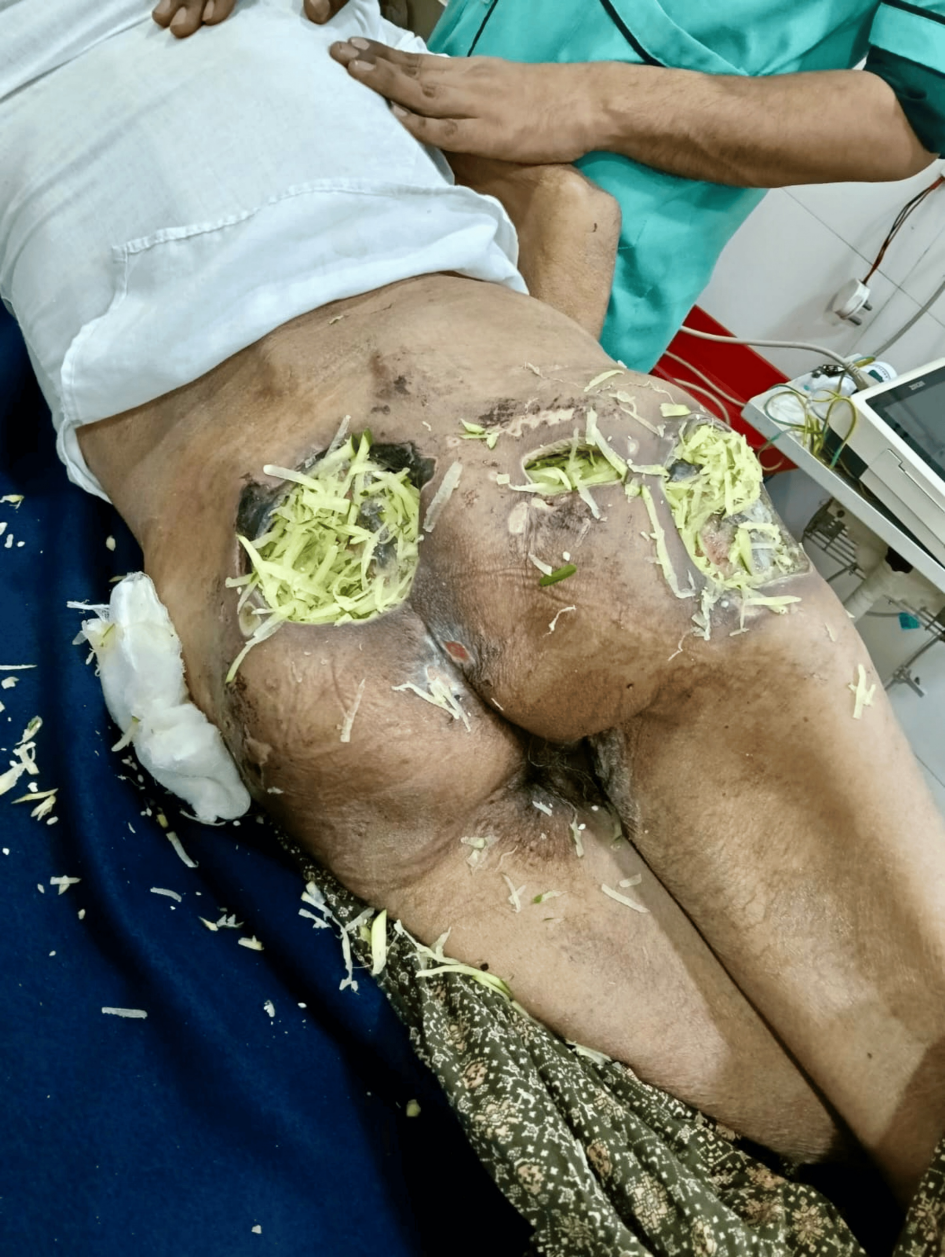
Ulcers after the application of freshly grated unripe papaya to the wound cavities, prior to the placement of vinegar-soaked gauze

Given the poor response and lack of access to advanced wound care materials, a novel dressing protocol was initiated. After thorough irrigation with sterile saline, freshly grated unripe papaya, prepared aseptically after washing, peeling, and grating, was applied directly into the wound cavities (Figure 2). The papaya-filled wounds were then covered with sterile gauze soaked in 5% white vinegar and secured in place. This regimen was initially performed twice daily while necrosis and purulent discharge persisted, and later reduced to once daily as the wound beds improved.

**Figure 3:**
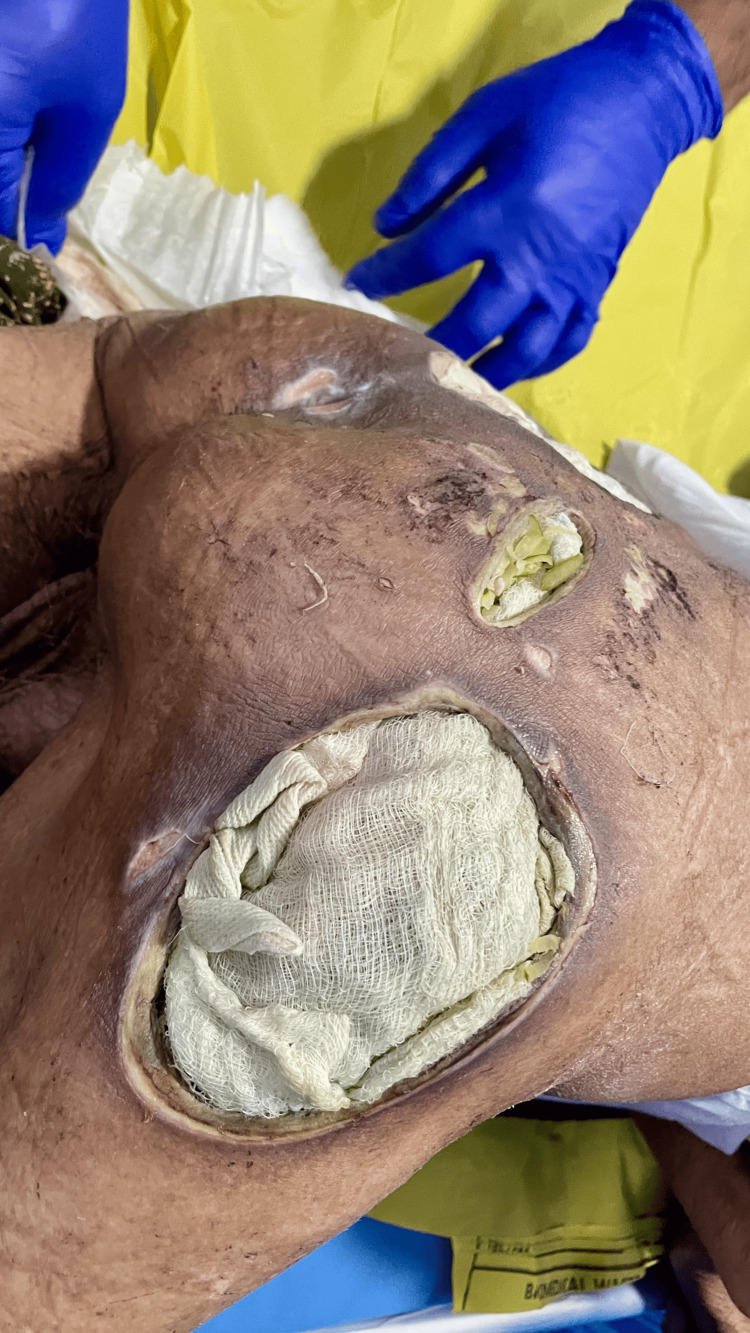
Day 3 of dressing with vinegar and papaya shows less greenish discoloration

By the third day of this regimen, foul odor had markedly reduced, and the gauze showed minimal greenish staining compared to baseline (Figure 3). Early signs of healthy granulation tissue were visible on wound inspection.

**Figure 4:**
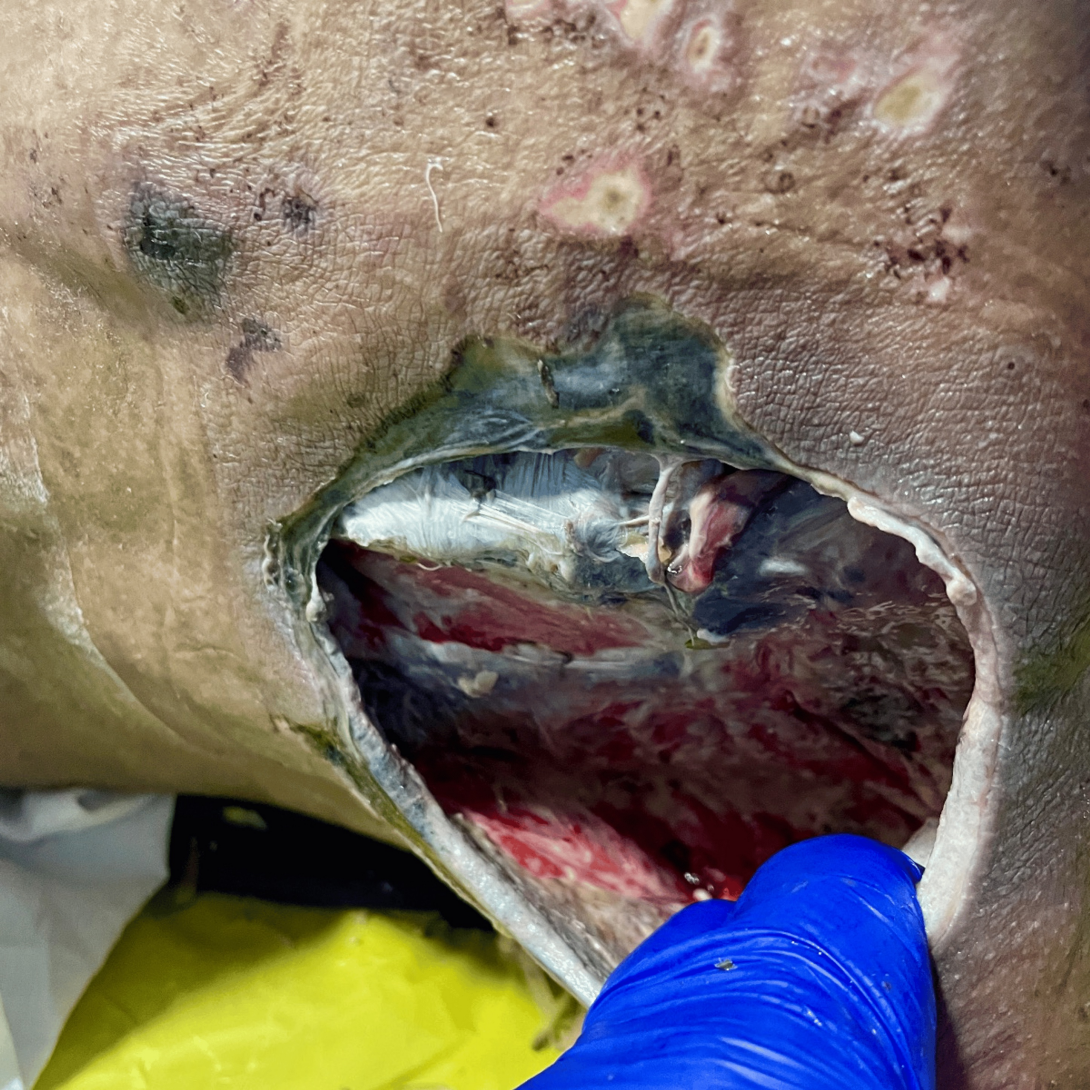
Day 10 of papaya–vinegar dressing showing near-complete clearance of slough, absence of greenish flakes, and a well-vascularized granulating wound bed

By the fifth day, approximately 60-70% of the slough had cleared, and the greenish discoloration had resolved. By days 7 to 10, the ulcers demonstrated near-complete slough clearance with a clean, well-vascularized granulating base, and significant improvement in odor (Figure 4).

At this stage, the ulcers developed a reddish, inflamed appearance, often described clinically as an “angry wound.” This represents the transition phase of wound healing, where papaya-vinegar dressings should be discontinued, as ongoing enzymatic debridement can cause further irritation. Saline dressings are recommended at this point to support epithelialization.

During treatment, the patient reported transient burning or painful sensations immediately after application of the vinegar-soaked gauze. This irritation was attributable to vinegar-induced stinging of exposed tissue, but it consistently subsided within a few minutes without the need for analgesia and diminished with subsequent dressings. No allergic reactions or sustained adverse effects were noted.

During this period, no additional nutritional supplementation or repositioning protocols were introduced, and the patient continued to be nursed on a water bed as before. Thus, the observed improvement was attributable primarily to the dressing intervention itself.

Unfortunately, shortly after these encouraging local improvements were observed, the patient died at home. The exact cause of death could not be confirmed, but given his advanced Parkinsonian disease, profound immobility, and general frailty, it was presumed to be unrelated to the wound itself. His passing precluded long-term follow-up and additional photographic documentation.

## Discussion

The management of pressure ulcers complicated by Pseudomonas aeruginosa infection remains difficult because of the organism’s ability to form biofilms, exhibit multidrug resistance, and produce malodor and greenish exudate that interfere with healing [2,3]. In many parts of the world, advanced wound care products, such as silver-impregnated dressings, hydrocolloids, and negative pressure wound therapy, are costly or inaccessible, leaving clinicians reliant on more basic measures [4]. Within this context, inexpensive and locally available agents, such as papaya and vinegar, hold promise as practical alternatives.

In our patient, the combination of freshly grated unripe papaya and vinegar-soaked gauze resulted in rapid improvement when conventional povidone-iodine dressings had failed. Odor reduction was noted by the third day, slough clearance reached nearly two-thirds by day 5, and by day 10, the ulcers demonstrated a clean, vascularized wound bed with healthy granulation.

These short-term outcomes are comparable to those reported in controlled studies of acetic acid dressings. Madhusudhan demonstrated in a randomized trial that 1% acetic acid achieved microbiological clearance of Pseudomonas in chronic wounds in a mean of 4.5 days, compared with 11-15 days with saline [7]. Sloss et al. reported the elimination of Pseudomonas in 14 of 16 burn and soft tissue wounds treated with daily 0.5-5% acetic acid dressings, most within 2 weeks [8]. Similarly, Ryssel et al. confirmed that 3% acetic acid significantly reduced bacterial load in infected burns [5]. Reviews emphasize that concentrations between 1% and 5% reliably inhibit Pseudomonas biofilms, although local stinging is common [9]. In our patient, brief burning was reported after the first few applications of vinegar-soaked gauze but subsided spontaneously, consistent with these prior findings. The rapid odor resolution and disappearance of greenish exudate in our case align closely with the antibacterial performance of acetic acid documented in prior studies. Notably, we used 5% household vinegar, a higher concentration than in most studies, which underscores both the feasibility and the need for further dose-finding research.

At the same time, the marked reduction in slough and appearance of granulation tissue within one week correspond well with the enzymatic debridement properties of papaya. Murthy et al. reported that papaya dressings achieved granulation significantly faster than hydrogen peroxide (2.5 vs. 6.2 days) [10]. Vasuki et al. conducted a randomized trial of 128 patients with chronic wounds and found papaya superior to saline, with significantly greater slough clearance and granulation by weeks 3 to 4 (p < 0.001 and p = 0.0082), although complete closure rates at 3 months were similar (78% vs. 72%) [11], while Starley et al. described successful use of papaya in pediatric burns, where it facilitated desloughing, reduced odor, and prepared wounds for grafting [6]. Mechanistic studies confirm that papain and chymopapain degrade necrotic tissue and promote healthy granulation, while experimental models demonstrate antioxidant and anti-inflammatory activity [12,13].

These findings are further supported by the Wound Healing and Management (WHAM) evidence summary, which reviewed multiple randomized and prospective studies of papaya pulp in wound care, including trials in chronic wounds, diabetic foot ulcers, and burns. It highlighted that there is no standardized method of papaya-pulp preparation, that enzymatic activity is greatest in raw or semi-ripe fruit, and that papaya-based debridement should be discontinued once slough has been cleared [14]. Our observation of rapid short-term improvement mirrors these findings and aligns with our deliberate use of freshly grated unripe papaya.

The unique aspect of this case lies in the concurrent application of papaya and vinegar, which, to our knowledge, has not been described previously in the management of infected pressure ulcers. Papaya has been studied across burns, diabetic ulcers, and postoperative wounds, while acetic acid has been validated in burns and chronic wounds, particularly against Pseudomonas. Used together, the two agents appeared to provide complementary benefits, rapid microbial suppression by vinegar and enzymatic debridement by papaya, producing a synergistic effect that may explain the unusually rapid progression from a sloughy, malodorous ulcer to a clean granulating wound bed in just 10 days. Most published reports using either agent alone describe comparable changes but often over a longer timeline, suggesting that this novel combination may accelerate healing beyond what each component achieves individually.

A further contribution of this case is the emphasis on wound-stage specificity. By day 10, the ulcers exhibited a reddish, inflamed appearance with robust granulation, often described clinically as an “angry wound.” At this stage, continuing papaya-vinegar risked excessive irritation due to ongoing enzymatic activity. By discontinuing the regimen and reverting to saline dressings, we aligned therapy with the wound’s natural progression and supported epithelialization. This adjustment has not been highlighted in prior literature but may represent a crucial practical lesson for clinicians using enzymatic debriders.

This report has several limitations. First, it describes a single case and therefore cannot establish efficacy or generalizability. Second, no standardized wound assessment scales, such as the Pressure Ulcer Scale for Healing (PUSH) or Bates-Jensen Wound Assessment Tool (BWAT), were employed. Third, no repeat cultures were obtained to confirm microbial clearance after treatment. Finally, the patient’s death shortly after wound improvement precluded long-term follow-up.

Despite these limitations, this case demonstrates that combining vinegar and papaya may offer a practical, low-cost option for managing infected pressure ulcers, particularly those colonized by Pseudomonas aeruginosa. The rapid reduction in odor, clearance of slough, and appearance of healthy granulation tissue suggest potential for wider application. Further prospective trials are needed to validate its efficacy, identify optimal protocols, and define the appropriate stage of use in wound healing.

## Conclusions

This case illustrates that daily dressings with freshly grated unripe papaya covered by vinegar-soaked gauze can provide rapid improvement in Pseudomonas-infected pressure ulcers, with reduction in odor, clearance of slough, and early granulation. The approach is inexpensive and practical for resource-limited settings, but should be stopped once the wound enters the inflamed healing phase. Further studies are required to validate its wider clinical use.
